# Supplementary figures and images for: Optimizing Outcomes through a Multidisciplinary Team Approach in Endometrial Cancer
Source: Healthcare (Basel). 2023 Dec 27;12(1):64. doi: 10.3390/healthcare12010064 (PMC10778853; doi:10.3390/healthcare12010064)

Supplementary Table S1.

|       | MDT |      |     |      |       |
|-------|-----|------|-----|------|-------|
|       | Yes |      | No  |      | Total |
|       | n.  | %    | n.  | %    |       |
| 2013  | 24  | 7.5  | 56  | 17.3 | 80    |
| 2014  | 20  | 6.3  | 62  | 19.1 | 82    |
| 2015  | 20  | 6.3  | 52  | 16.0 | 72    |
| 2016  | 44  | 13.8 | 42  | 13.0 | 86    |
| 2017  | 48  | 15.0 | 23  | 7.1  | 71    |
| 2018  | 57  | 17.9 | 31  | 9.6  | 88    |
| 2019  | 54  | 16.9 | 42  | 13.0 | 96    |
| 2020  | 52  | 16.3 | 16  | 4.9  | 68    |
| Total | 319 | 100  | 324 | 100  | 643   |

Supplementary Figure S1

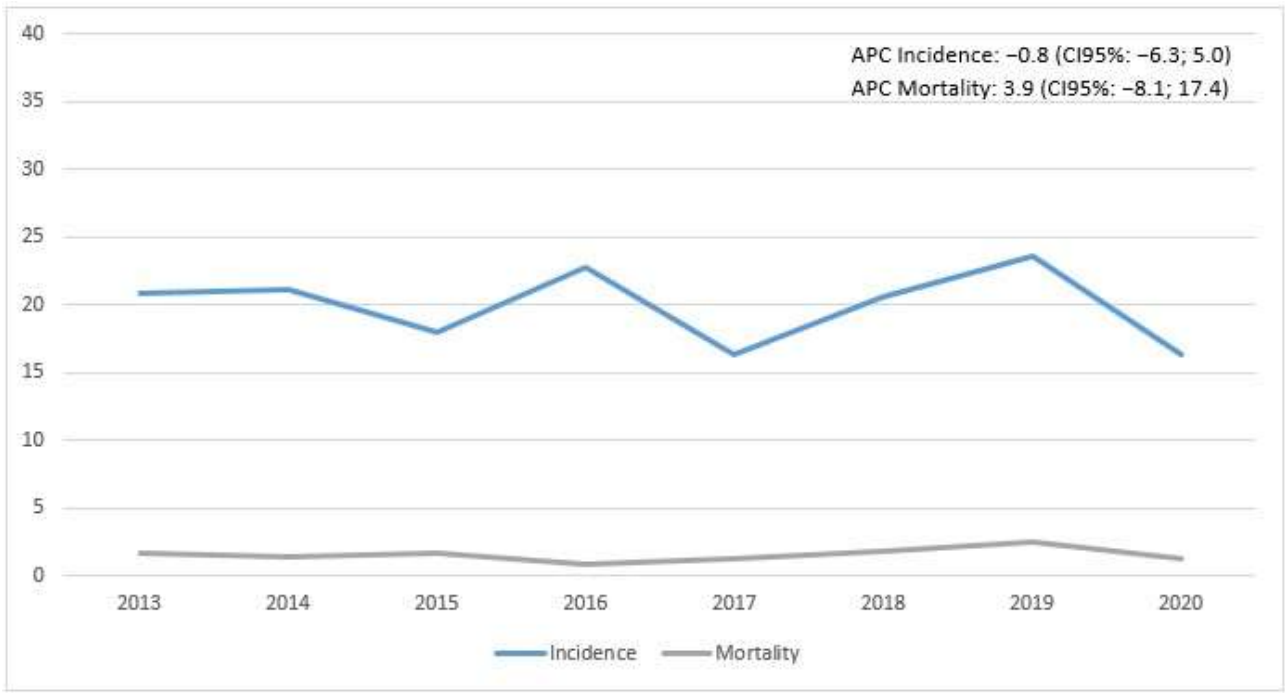

Supplement: Supplementary file 1 [file healthcare-12-00064-s001.zip › healthcare-2760433-supplementary.pdf]
